# Molecular events in gastric carcinogenesis

**Published:** 2014-09-25

**Authors:** C Mahu, AP Purcarea, CM Gheorghe, MR Purcarea

**Affiliations:** * "Prof. Dr. D. Gerota" Ministry of Internal Affairs Emergency Hospital, Bucharest, Romania; ** "Carol Davila" University of Medicine and Pharmacy, Bucharest, Romania; *** "Dr. Carol Davila" Clinical Nephrology Hospital, Bucharest, Romania

**Keywords:** gastric carcinogenesis, molecular events

## Abstract

Abstract

Gastric cancer represents an important problem for the public health, being one of the main causes of mortality. At present, it represents the second cause of mortality due to cancer, after the bronchopulmonary cancer in men and the fourth cause of mortality in women.

Important progresses have been made in the last couple of years in determining the neoplastic etiopathogenesis, but it cannot be affirmed that the genetic mutations chain, which leads to the appearance of the malignant cell, has been fully understood.

Gastric cancer represents an important problem for the public health, being one of the main causes of mortality. At present, it represents the second cause of mortality due to cancer, after the bronchopulmonary cancer in men and the fourth cause of mortality in women [**1**]. 

Important progresses have been made in the last couple of years in determining the neoplastic etiopathogenesis, but it cannot be affirmed that the genetic mutations chain, which leads to the appearance of the malignant cell, has been fully understood. 

 Two of the essential characteristics of the cancer cells are the uncontrolled growth and the capacity to metastasize. The malignant phenotype of a cell represents the result of a series of genetic modifications, which remove the cellular growth restriction mechanisms and induce new characteristics, which give the cell the capacity of metastasizing, these being the surface receptors, enzymes, cytokines and angiogenic factors. These genetic modifications usually imply the expression the abnormal or exacerbated activity of some genes, proto oncogenes (usually growth factors or their receptors, the enzymes in the growth cycles or the transcription factors). These genetic modifications take place by punctiform mutations, gene amplification, gene rearrangement or epigenetic modifications (the alteration of the gene methylation). 

 The gastric carcinogenesis process, through which the normal mucosa turns to cancer, probably implies many risk factors, some of them intervening in a precocious stage, some of them even later.

 The gastric carcinomatosis represents a pluri-factorial process [**2**].

 Based on the study regarding the natural evolution of chronic gastritis, starting from the superficial gastritis to atrophic gastritis, intestinal metaplasia and the preneoplastic lesions (Korea model), the implication of H. pylori in carcinogenesis is based on the fact that this infection represents the main cause of chronic gastritis. A special role in this process is given to H. Pylori cag.A positive roots, whose virulence can lead to the appearance of a severe inflammation of the gastric mucosa, being, the common element in patients suffering from gastric cancer [**3**].

 From the histologic point of view, gastric cancer falls into two categories, intestinal type and diffuse type of cancer. Approximately 50% of the gastric cancer cases are diffuse, most of them occurring in cases in which H. pylori gastritis is not atrophic and metaplasic [4,5]. Typically, diffuse gastric cancer is characterized by a tumor growth and invasion with isolated, weakly differentiated or undifferentiated cells. 

Tahara hypothesis has been incriminated – the key being c-met gene, which encodes c-met protein. 

** Cell markers in the cancer process**

 The gastrointestinal carcinogenesis is considered to reflect a process with many stages of morphological modifications, being accompanied by a progressive accumulation of the genetic modifications [**6**]. Taking into account the data accumulated until present, which refer to the cellular molecular events in gastric cancer, it is important to notice if there is one or more pathogenic ways that lead to gastric carcinoma [**7**]. Cardia neoplasms do not seem to be connected with the positive H. pylori infection in comparison with the tubular, papillary and mucinous (WHO classification) types of gastric cancer, which are considered synonymous with the intestinal type (Lauren classification), "signet ring" cells carcinoma, undifferentiated forms (WHO classification) being interpreted as diffuse tumors (Lauren). Relatively recent data underline the involvement of positive H. pylori cag.A roots in the carcinogenesis process (revealing the importance of the duration of infection as a determinant factor in gastric carcinogenesis).

 The bacterial density has an important role in the tumor initiation phase [**8**]. 

The local production of alpha (TNF-) tumor necrosis factor and of interlukin-8 by the surface epithelial cells in cardiac gastritis, determines an important inflammatory reaction, including the mono- and polynuclear types, macrophages, T and B-lymphocytes.

In the case of H. pylori infection, there is a growth in the rate of gastric epithelial cells proliferation, with the appearance of chronic gastritis and a raise in the vulnerability of the epithelial cells to mutagens. Currently, it is considered that approximately 50% of the H. pylori infected patients develop atrophic gastritis. Of these, 80% (meaning 40% of the total infected population) develop intestinal metaplasia and 20% or 80% of the total population develop type III intestinal metaplasia or low degree dysplasia. Approximately 10-20% of these or 0,8-1,6% of the total will develop gastric cancer. As a result, there is a model (similar to the Markov model of "unprocessed selection") through which, the positive H. pylori subjects are estimated to have a gastric cancer risk [**9**].

**The proliferation and apoptosis in gastric carcinogenesis**

 The raised cells proliferation represents a usual observation in preneoplasia and neoplasia. According to the model proposed by Ames and col. Cit. de Moss SF [**6**], the cells proliferation predisposes to cancer by raising the chance of appearance of somatic mutations. 

 The modifications in the genomic establishment and the mutations or the modifications in the tumor genome can appear long before the appearance of the preneoplastic or obvious neoplastic lesions, affirmations which are sustained by a series of events: abnormal synthesis of mucus glycoproteins (Lewis blood type, CA-19-9, Sialy Le(x), etc.) and the abnormal expression of K-ras gene in the case of patients with chronic gastritis or intestinal metaplasia. More recent conceptions regarding carcinogenesis underline that this uncontrolled proliferation, characteristic to cancer, is not owed only to the raised number of cells but also to a relative deficiency, which intervenes in the programmed death of the cells (apoptosis) in gastric cancer [**10**]. Studying the pieces of gastric resection, there is a difference between the values of the apoptotic index, registered at the level of the well-differentiated tumors, compared to the weakly differentiated ones. It was demonstrated that there is a raise in the rate of gastric epithelial cells proliferation in preneoplastic stages, and recently, also in chronic gastritis associated to H. pylori infection.

The relationships between the cellular proliferation activity in gastric cancer and the normal epithelium can be studied by flux cytometry technique, the activity of the ornithine decarboxylase enzyme or by a quantitative determination of the nucleolar organizer regions (AgNORs), an indirect marker of proliferation. 

**Molecular processes involved in gastric carcinogenesis**

**P53 gene**

 The mutation of p53 gene is one of the most common anomalies in human cancer, probably due to the main role of this gene in regulating the cycle of the normal cell. The anomalies of p53 gene, described in human cancer are usually punctiform mutations or allelic deletions, which will lead to the loss of p53 gene, so that this "guardian of the genome" cannot activate the protection paths that intervene in stopping the cycle of the cell and the apoptosis.

 Using the immunohistochemistry and PCR-SSCP, the mutations of p53 gene have been detected in approximately 50% of the advanced gastric cancers. It was highlighted that in diffuse gastric cancers, the mutations of p53 gene intervene in a late stage [**6**]. Some studies show that the mutations of p53 gene have also been identified in gastric cancer with metastases in a percent of 77% [**11**]. Generally, it is considered that p53 accumulation is correlated with the presence of ganglionar metastasis and with a significantly reduced survival rate [12,13]. Modifications of p53 have been found in severe dysplasia patients or precocious, intestinal or diffuse gastric cancer. All these findings have suggested the fact that highlighting the p53 anomalies can contribute to the histological recognizing of high degree dysplasia in gastric biopsies, especially in the cases of active or erosive gastritis, in which the differentiation between the regenerative epithelial hyperplasia and the high degree dysplasia can be difficult to achieve. 

 It is not known yet if H. pylori and/ or the associated inflammatory reaction can produce p53 mutations or if H. pylori infection and these mutations of p53 are synergic events in gastric carcinogenesis. 

**TGF-beta and the instability of the microsatellites**

 The TGF-beta family (transforming growth factor) contains polypeptide dimers which are disulfide bonded, structurally related and which affect the propagation, differentiation, apoptosis and interaction of the cells with the cellular matrix [**14**]. Three types of TGF-beta receptors have been identified: TGF I, involved in fibronectin synthesis, having effects on the extracellular matrix; 

 -TGFR II is the main receptor involved in the antiproliferative effect, also in the apoptotic one and the phosphorylation of the retinoblastoma protein; 

 -TGFR III is not involved in the transduction of signals that are TGF-beta mediated. 

 TGF-beta is produced by gastric epithelial cells, including the cellular lines of gastric cancer and intervenes both in inhibiting the growth and in provoking the apoptosis, after stopping the cellular cycle in GI/S stage, even if the action mode is still unclear. It is assumed that there is an involvement of c-myc and a reduction in expressing the cyclins, meaning cyclin A and the kinase which is dependent on cdk2 cyclin. 

 Most of TGFR II mutations can be secondary to the microsatellites’ instability (short and repeated nucleotide sequences that can be found at the level of the genome) [**15**]. 

 The microsatellites’ instability can appear very soon in the tumoral process. There are uncertainties connected to the microsatellites’ instability: it is limited only to certain parts of the stomach or it is connected to p53 mutations, APC mutations or ras expression. The microsatellites’ instability has been absent in the normal gastric mucosa, compared with the instability seen in 43% of the intestinal metaplasia cases and 67% of the carcinomas. 

** Cyclins**

 Represent a family of proteins through which the interaction with the kinases that depend on cyclin (CDKs) regulates the evolution of cells during the cellular cycle. The mutation and the excessive manifestation of cyclin genes have been discovered in many human cancer types. The growth of expressing DI cyclin has been noticed in almost half the total amount of gastric cancers located at the level of the body, as well as the cardia, most frequently the intestinal type than the diffuse type. Certain proteins which are capable of inhibiting the kinases that depend on cyclins, including p15, p16, p21 (cip1/waf1) and p27 have been identified. These cdk inhibitors are involved in regulating the cellular cycle and can have properties similar to the tumor suppressor genes.

 It is considered that subsequent researches on cyclins, kinases dependant on cyclin and their inhibitors can help in studying the way H. pylori influences the proliferation and the apoptosis of the epithelial cells. 

**Tumor suppressor genes**

** E-cadherin-catenin complex**

 E-cadherin has an important role in the adhesion of the gastric epithelial cells; it interacts with the intracellular cytoskeleton through the molecules of the cytoplasm, which are called catenins (alpha, beta, gamma). Generally, in gastric cancer and particularly in the diffuse type of cancer, a reduction of the level of E-cadherin has been noticed [**16**]. 

 DCC gene-is a gene discovered in colon cancer, the tumor suppressor gene situated at the level of 18q21 chromosome. The loss of heterozygosity to 18q has been noticed in 61% of the gastric cancers, including most of the precocious gastric ones [**4**]. 

 APC gene 

 The mutations of APC gene (adenomatosis polyposis coli) and MCC (mutated in colon cancer), located on 5q chromosome, can be detected in approximately 64% of the cases.

 K-ras oncogene 

 The activity of K-ras oncogene, as well as c-erb and the AD3 abnormal transcription can be common events in gastric cancer. 

 Retinoblastoma protein 

 It is a 105kDa protein, which, in inactive or hypophosphorylated stage, determines the suppression in the evolution of G1 cell cycle, through the connection with E2F transcription factor. Recent studies, which examine the expression of the retinoblastoma in the stomach through immunohistochemistry, noticed the expression of this protein in the normal proliferation of the cells of the gastric epithelium, but also the growth in the proliferative areas in chronic gastritis or the ones with intestinal metaplasia, in dysplasia and gastric cancers, probably reflecting the cellular proliferation [**6**]. 

**Peptides growth factors in gastric carcinogenesis**

 The normal development and the cells’ differentiation in the gastrointestinal tract is under the influence of autocrine and paracrine secretion of the growth factors specific to peptides which are responsible for the control of maturation, differentiation and apoptosis. Generally, the stomach peptides growth factors interact with the receptors at the surface of the cells and activate the cytoplasmic tyrosine-kinase. 

** The growth epidermal factors family**

 The members of EGT family are mitogens in most of the tissues, interceding their effects after the connection with the specific receptors, the EGF receptor (type I EGF receptor) and the type II EGF receptor, also known as c-erb-B2 (Her2-neu, p185 c-neu). Probably, the stomach does not synthesize the EGF, the main source being the salivary glands. TGF-alpha, the main EGF-like growth factor produced by the normal stomach is mainly synthesized by the package of mucosal cells. The TGF-alpha immunoreactivity is present in intestinal metaplasia and also in the intestinal type of the diffuse types of cancers [**17**]. 

**The fibroblastic growth factor family**

 Little is known at present regarding these growth factors in cancer, with the exception of k-sam gene, which codes a receptor of the fibroblast growth factors. The amplification of this gene can appear in approximately 20% of the diffuse types of gastric cancers [**18**]. 

 Telomerase. The activity of telomerase has been noticed in a significant number of cases of gastric cancer. 

 Homeobox genes. In the last couple of years, different studies have been highlighting the importance of Cdx-1 gene in the development of intestinal metaplasia but not in the next stages of gastric carcinogenesis. 

 In conclusion, gastric carcinoma appears, from the biological point of view, as a heterogeneous disease that implies numerous genetic and epigenetic alterations, such as E-cadherin (CDH1) gene specific mutations, mutations of the gene at the level of p53 protein [**18**]. Moreover, a significant amount of gastric carcinomas is demonstrated by Runx3 loss due to the hemizygous deletion and the hypermethylation of the promoter region. 

 The epigenetic changes can be attractive targets for the treatment of modulators cancer.
